# Peptides Evaluated In Silico, In Vitro, and In Vivo as Therapeutic Tools for Obesity: A Systematic Review

**DOI:** 10.3390/ijms25179646

**Published:** 2024-09-06

**Authors:** Ana Júlia Felipe Camelo Aguiar, Wendjilla Fortunato de Medeiros, Juliana Kelly da Silva-Maia, Ingrid Wilza Leal Bezerra, Grasiela Piuvezam, Ana Heloneida de Araújo Morais

**Affiliations:** 1 Biochemistry and Molecular Biology Postgraduate Program, Biosciences Center, Federal University of Rio Grande do Norte, Natal 59078-970, RN, Brazil; anajulianutri@gmail.com; 2 Nutrition Postgraduate Program, Center for Health Sciences, Federal University of Rio Grande do Norte, Natal 59078-900, RN, Brazil; wendjillanutri@gmail.com (W.F.d.M.); juliana.maia@ufrn.br (J.K.d.S.-M.); 3 Department of Nutrition, Center for Health Sciences, Federal University of Rio Grande do Norte, Natal 59078-900, RN, Brazil; ingrid.bezerra@ufrn.br; 4 Health Sciences Postgraduate Program, Center for Health Sciences, Federal University of Rio Grande do Norte, Natal 59078-900, RN, Brazil; gpiuvezam@yahoo.com.br; 5 Public Health Department, Federal University of Rio Grande do Norte, Natal 59078-970, RN, Brazil

**Keywords:** anti-obesity agent, obesity management, computer simulation, molecular dynamics simulation, molecular docking, peptides, molecular conformation, obesity

## Abstract

Bioinformatics has emerged as a valuable tool for screening drugs and understanding their effects. This systematic review aimed to evaluate whether in silico studies using anti-obesity peptides targeting therapeutic pathways for obesity, when subsequently evaluated in vitro and in vivo, demonstrated effects consistent with those predicted in the computational analysis. The review was framed by the question: “What peptides or proteins have been used to treat obesity in in silico studies?” and structured according to the acronym PECo. The systematic review protocol was developed and registered in PROSPERO (CRD42022355540) in accordance with the PRISMA-P, and all stages of the review adhered to these guidelines. Studies were sourced from the following databases: PubMed, ScienceDirect, Scopus, Web of Science, Virtual Heath Library, and EMBASE. The search strategies resulted in 1015 articles, of which, based on the exclusion and inclusion criteria, 7 were included in this systematic review. The anti-obesity peptides identified originated from various sources including bovine alpha-lactalbumin from cocoa seed (*Theobroma cacao* L.), chia seed (*Salvia hispanica* L.), rice bran (*Oryza sativa*), sesame (*Sesamum indicum* L.), sea buckthorn seed flour (*Hippophae rhamnoides*), and adzuki beans (*Vigna angularis*). All articles underwent in vitro and in vivo reassessment and used molecular docking methodology in their in silico studies. Among the studies included in the review, 46.15% were classified as having an “uncertain risk of bias” in six of the thirteen criteria evaluated. The primary target investigated was pancreatic lipase (n = 5), with all peptides targeting this enzyme demonstrating inhibition, a finding supported both in vitro and in vivo. Additionally, other peptides were identified as PPARγ and PPARα agonists (n = 2). Notably, all peptides exhibited different mechanisms of action in lipid metabolism and adipogenesis. The findings of this systematic review underscore the effectiveness of computational simulation as a screening tool, providing crucial insights and guiding in vitro and in vivo investigations for the discovery of novel anti-obesity peptides.

## 1. Introduction

The rapid evolution of modern society, characterized by heightened socioeconomic advancement and urbanization, has facilitated the emergence of an obesogenic lifestyle among the populace, marked by sedentary habits and excessive consumption of highly processed foods rich in fats and sugars [1].

According to the World Health Organization—WHO (2023) [2], global projections indicate that by 2035, more than 4 billion people will be overweight and obese (BMI ≥ 25 kg/m^2^), compared to over 2.6 billion in 2020. This represents an escalation from 38% of the world population in 2020 to over 50% by 2035 (excluding children under 5 years old). The estimative is that obesity (BMI ≥ 30 kg/m^2^) alone will surge from 14% to 24% of the population within the same time frame, affecting nearly 2 billion adults, children, and adolescents. The rise in obesity is expected to be particularly pronounced among children and adolescents, increasing from 10% to 20% in boys and from 8% to 18% in girls worldwide [2].

Hence, obesity has increasingly become a global public health priority worldwide due to its increasing prevalence and its correlation with numerous health complications, establishing itself as one of the most common chronic non-communicable diseases (NCDs) [3,4].

Treatment for obesity encompasses interventions from five main categories: behavioral modifications, dietary changes, physical activity, pharmacotherapy, and ultimately, surgical intervention. Lifestyle modifications, especially physical exercise and the adoption of a balanced and healthy diet, form the foundation for obesity therapy [2].

Perspectives studies indicate that behavioral modifications targeting lifestyle habits can lead to weight reduction ranging from 5–10% [3,5,6,7]. Sustaining the achieved weight is the biggest challenge, prompting clinical guidelines to advocate for the extended use of anti-obesity medications when lifestyle modifications alone prove insufficient for achieving or maintaining weight loss [5,8].

Currently, six medications have received approval from the Food and Drug Administration (FDA) for the long-term treatment of obesity. These include semaglutide and liraglutide (glucagon-like peptide 1 GLP-1 receptor agonists), tirzepatide (an insulinotropic polypeptide dependent on glucose/GLP-1 agonist), orlistat, phentermine-topiramate, and naltrexone-bupropion [5,8,9].

Surgical interventions include endoscopic procedures such as intragastric balloon and endoscopic gastroplasty, as well as metabolic surgeries like laparoscopic sleeve gastrectomy and Roux-en-Y gastric bypass, which demonstrate the most significant weight loss outcomes [8,9]. It is well-established that obesity is governed and maintained by a central dysregulation of the appetite/satiety mechanism. Furthermore, a counter-regulatory response to a negative energy balance can contribute to weight regain [6,10].

Given the complexity of treating obesity and the potential side effects associated with drug therapy and/or surgical interventions, research into alternative treatment methods have been expanding worldwide. Particularly, there is growing interest in exploring bioactive compounds and nutraceuticals of natural origin due to their anti-obesity properties [11,12]. However, interpreting study findings remains challenging, primarily because much of the data stems from in vitro and animal research. Despite the encouraging results, questions persist regarding the mechanism of action and molecular functionality [11].

Therefore, a recent approach that has been used to minimize the cost and time of the process of developing new drugs and understanding their action at a molecular level is computer-aided drug design (CADD). This methodology poses a significant challenge in the pharmaceutical industry, where extensive time and financial resources are required to go through all phases of development [13].

With the advancement of bioinformatics and technological progress, computational studies have emerged as a pivotal component in scientific research. These studies employ tools to unravel molecular structures, interactions between molecules, and explore possible biological actions, thereby playing central roles in the global ecosystems of biological and biomedical research and education [14]. This dual function serves to elucidate molecular dynamics and identify novel therapeutic alternatives for globally significant diseases like obesity.

Consequently, the integration of in silico and experimental studies has become a strategy approach in drug discovery. This allows for the testing of initial hypotheses using computational tools, accelerating the general process of identifying proteins and peptides, reducing cost and time [15]. Bioactive peptides and proteins have high potential to treat various diseases with specificity and biological safety. Furthermore, protein-peptide and protein-protein interactions perform essential activities in many biological functions underscoring the escalating interest in and pursuit of peptide-based drug development [16].

The search for bioactive proteins and peptides targeting disease-relevant receptors typically requires high-throughput screening methods, with computational studies being widely explored for their cost-effectiveness and efficacy [16,17]. Virtual screening of protein-protein and protein-peptide poses a complex challenge due to the inherent characteristics of these ligands, including their considerable conformational plasticity. This directly impacts the identification and optimization processes in protein-based drug design projects [15,16,17].

Given the public health challenge that obesity presents, along with the complexities of treatment and potential side effects associated with medications and/or surgical/metabolic interventions, it is imperative to search for new therapeutic alternatives [2,3,4,11,12]. Computational simulation studies offer a promising avenue for the discovery for proteins and/or or natural bioactive peptides aimed at treating obesity [15]. 

In recent years, several scientific studies have focused on the delivery of naturally-derived bioactive peptides as specific potential foods and as lead compounds in drug discovery, while also gaining insights into their roles in health effects [18,19,20,21]. These peptides have demonstrated a positive impact on various biological functions, including antioxidant, antidiabetic, antihypertensive, and antimicrobial properties. Additionally, there is a wide range of natural protein sources that can serve as basis for generating these peptides [22,23,24,25].

Thus, the systematic review aimed to evaluate whether in silico studies using anti-obesity peptides targets therapeutic pathways for obesity, when subsequently evaluated in vitro and in vivo, demonstrated effects consistent with those predicted in the computational analysis. Additionally, the review encompassed studies examining proteins and/or peptides of natural origin that underwent in silico evaluation for potential obesity treatment, followed by subsequent in vivo and/or in vitro reassessment.

## 2. Methods

### 2.1. Protocol and Registration

The systematic review (SR) was preceded by the publication of the protocol in a scientific journal by Medeiros et al. [26], following the guidelines described in Preferred Reporting Items for Systematic Reviews and Meta-Analyses Protocols (PRISMA-P) [27] and registered in the International Prospective Register of Systematic Reviews (PROSPERO) under the registration number CRD42022355540 with the following access link: https://www.crd.york.ac.uk/prospero/display_record.php?ID=CRD42022355540. 

Initially this review included computational studies addressing the research question “Which peptides or proteins have been used to treat obesity in in silico studies?”, as described in the SR protocol article [26]. However, during the initial stages of the SR search, it became apparent that restricting the peptides and/or proteins to those of natural origin was necessary to enhance therapeutic agent homogeneity. Furthermore, only studies that conducted in vivo or in vitro reassessment of the same peptide and/or protein tested in the computational model were included, with the aim of considering the in silico findings in the context of potential clinical applications for obesity treatment. 

All modifications were documented in PROSPERO [26]. This study is exempt from evaluation by the Research Ethics Committee (REC).

### 2.2. Search Question

The research question guiding this SR was: “Which peptides or proteins have been used to treat obesity in the in silico studies?”; it was structured according to the acronym PECo (P, problem; E, exposure; Co, context) (Table 1). 

### 2.3. Inclusion Criteria

This SR included original computer simulation research conducting molecular dynamics and/or docking studies involving interactions between proteins and/or peptides of natural origin and therapeutic targets relevant to obesity treatment. Only studies were included where the same proteins and/or peptides underwent re-evaluation in in vivo and/or in vitro experimental models.

### 2.4. Exclusion Criteria

Studies exclusively in vivo, in vitro, or in silico without re-evaluation in vivo and/or in vitro, as well as those with proteins and/or peptides of synthetic origin, guidelines, review articles, theses, dissertations, letters, conference abstracts, gray literature, preprint, and Data In Brief were excluded from this review. Additionally, studies that did not employ molecular dynamics (DM) and/or molecular docking were also excluded, as well as those focusing on other comorbidities or diseases other than obesity.

### 2.5. Search Strategies

A systematic literature search was conducted in June 2024 utilizing the following electronic databases: PubMed (https://pubmed.ncbi.nlm.nih.gov/ accessed on 30 June 2024); ScienceDirect (https://www.sciencedirect.com/ accessed on 30 June 2024); Scopus (https://www.scopus.com/ accessed on 30 June 2024); Web of Science (https://www.webofscience.com/ accessed on 30 June 2024); Virtual Health Library (VHL) (https://bvsms.saude.gov.br/ accessed on 30 June 2024); and EMBASE (https://www.embase.com/ accessed on 30 June 2024). Additionally, a manual search was performed to identify relevant studies that were not captured by the electronic search strategies but could contribute to answering the research question.

The construction of the best search strategies was carried out seeking high sensitivity and using terms in a controlled vocabulary. Based on the research question, MeSH (Medical Subject Heading) health descriptors were selected in Medline and their respective “entry terms”. The terms selected to construct the search strategy were “protein”, “peptide”, “treatment”, “in silico”, “computer simulation”, “molecular dynamics simulation”, “molecular dynamics”, “molecular docking simulation”, “molecular docking”, “obesity”, and “obese”. The terms selected to compose the search strategy were combined with the Boolean operators of intersection “OR” and addition “AND”.

The research was carried out without imposing restrictions on language or publication date. Truncation symbols were also employed to enhance word variation. To identify the most suitable search equation for selecting articles relevant to the SR based on the research question, various strategies were tested, compared, and refined, considering the unique features of each database (Table 2).

### 2.6. Data Selection and Extration

**Figure 1:**
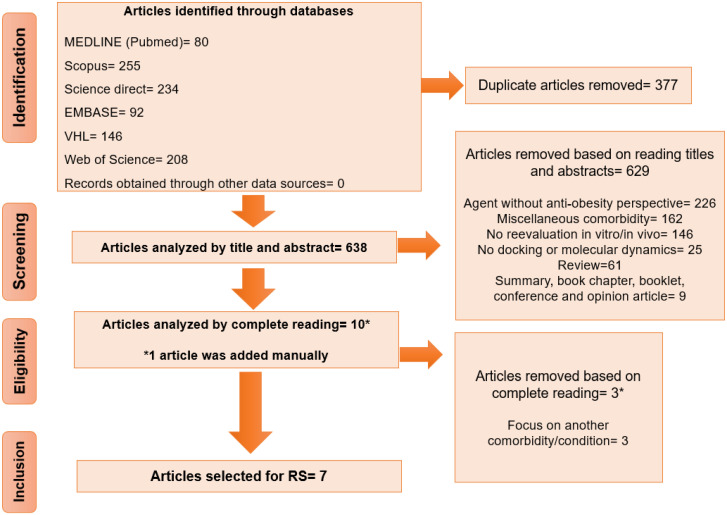
Flowchart of article selection adapted from the Preferred Reporting Items for Systematic Reviews (PRISMA). * = Article that was manually included.

The article selection was conducted using the Rayyan QCR84 software (version 0.1.0) [28]. Articles that were not identified through the search strategies but potentially addressed the research question could be manually included; however, no articles fitting the research question were found outside of the screened search results. All steps were performed by two independent reviewers, with a third reviewer available to resolve any discrepancies. Rayyan made it possible to delete duplicates and manage the following steps. First, the articles were screened based on title and abstracts, followed by full-text review of those selected in the initial stage for data extraction, with adherence to predefined inclusion and exclusion criteria throughout all stages. Excluded articles and their respective justifications were documented. A flowchart showing the article selection and exclusion process was presented using the flowchart in accordance with PRISMA (Figure 1). 

The studies selected for inclusion in the research had their data extracted by two independent reviewers. The extraction data included authors, model (in silico), technique used (docking and/or DM), interaction and main outcomes (type, protein source and/or peptide, peptide sequence, and other pertinent information such as type of inhibition, mass, hydrophobicity, isoelectric point, and risk prediction model), in addition to in vitro and/or in vivo effects, potential applications in obesity, and other relevant data. The extracted data from selected studies were organized in a predefined table using the Microsoft Excel^®^ program.

### 2.7. Data Analysis and Synthesis

The synthesis of the obtained data was accomplished through a narrative description. Descriptive tables were also prepared to present the extracted characteristics from the selected research, including pertinent information regarding study protocols and results, categorized according to each type of protein/peptide addressed. References and citations were made using Mendeley software (version 1.19.8) (https://www.mendeley.com/ accessed from June to 30 August 2024).

### 2.8. Risk of Bias and Assessment of Study Quality

After reading the articles included in the SR, the methodological quality of the studies was assessed. The analysis of the risk of bias and the quality of the studies will be carried out by a researcher, with the participation of a second evaluator, if necessary, to resolve disagreements. The risk of bias was assessed following the adapted checklist developed by Taldaev and collaborators [29] (Table S1), as there is currently no standardized tool available specifically designed for molecular dynamics and/or docking studies. To include the selected studies in this SR, the tool was adapted by adding an option for “in vivo” validation in the section regarding the verification of docking results by in vitro studies (Table S1). All evaluators were trained and calibrated to ensure consistent application of the tools.

## 3. Results and Discussion

### 3.1. Selection and Characteristics of Studies

To identify the most effective search strategies, adjustments were made to the different search equations (Table 2), followed by testing to determine the optimal approach for addressing the research question within each database. Initially, 1015 articles were identified, with duplicates subsequently excluded. Following title and abstract analysis, 638 articles remained for consideration, of which 10 were selected for full reading and 1 was added manually, as it had not been captured by any search strategy (Figure 1).

Following a thorough examination, 7 articles fulfilled the criteria for inclusion in the research. All selected articles were in English and were published between the years 2020 and 2024. A total of 54 peptides were identified, each possessing distinct amino acid sequences and originating from different natural sources, predominantly plants (Table 3). Thus, the procedures encompassing extraction, synthesis, data analysis, and assessment of the risk of bias were executed.

### 3.2. Bias Risk Assessment

The risk of bias (Table 2) in the studies was evaluated utilizing a checklist (Table S1) adapted from Taldaev et al. [29]. Regarding ligand selection, 85.75% of the studies reported the implementation of filtering steps, indicative of low bias. However, only 28.57% clearly mentioned the ligand ionization step, while 100% of studies indicated the generation of energetically feasible conformations for the selected ligands, thus demonstrating a low risk of bias in all studies in this regard.

Concerning the resolution of the experimental structures of the targets, 83.33% did not exceed 2.5 Å, thereby indicating a low risk of bias, since values above 2.5 Å indicate a high risk of bias. Furthermore, all (100%) of the studies were classified as having a low risk of bias regarding the method of obtaining the structure. This determination stems from the fact that all 3D structures of the targets were sourced from the Protein Data Bank (PDB), with none derived theoretically.

**Figure 2:**
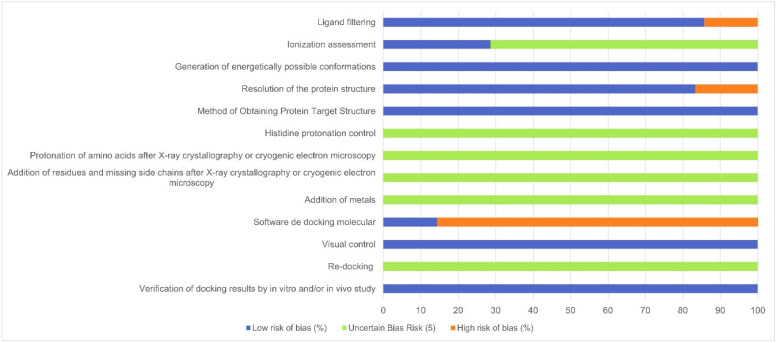
Graph of risk of bias of studies selected for SR (n = 7). Adapted from Taldaev et al. [29].

The aspects linked to target optimization exhibited the highest level of uncertain risk of bias (Figure 2), as none of the 7 studies detailed the procedures related to this aspect. The reason is probably the greater focus on ligands than targets within these studies, a distinction not explicitly articulated by the authors, but this has also been observed in a recent SR by Medeiros et al. [37].

Regarding the software utilized in the molecular docking study, only one study (14.29%) presented a low risk of bias. In the evaluation of the results, all (100%) studies received low risk in the visual control item. However, none of the studies reported whether re-docking was conducted, leading to an uncertain risk of bias in this regard. As for the last item assessed, pertaining to re-evaluation in in vitro/in vivo studies, all studies carried out reassessment, consequently being classified as low risk of bias.

The risk of bias analysis revealed that out of the 13 items, 6 (46.15%) presented an uncertain risk of bias. This underscores the imperative to enhance methodological rigor in computational studies employing bioinformatics to evaluate agents and targets for treating obesity. The absence of such data in studies complicates the assessment of methodological quality and implies reproducibility for future research.

Therefore, it is crucial to select and prepare ligands along with their corresponding targets to facilitate the assessment of methodological quality in development drug models based on structure–ligand relationships. This approach is essential for elucidating the pharmacological characteristics and mechanisms of action, ultimately aiding in the discovery of optimal therapeutic strategies [38].

### 3.3. Characteristics of Research, Extraction, and Synthesis of Study Data

#### 3.3.1. In Silico Studies of Anti-Obesity Peptides

Bioactive peptides typically consist of 2–20 amino acids (aa) and represent unique protein fragments that exert several beneficial effects on physiological functions, in addition to generally exhibiting low toxicity and antigenicity. These peptides can be obtained through enzymatic hydrolysis, fermentation, or gastrointestinal digestion. Currently, several studies are exploring bioactive peptides from various sources, including plant, animal, and microbial proteins [39,40]. 

Peptides are recognized as a source of amino acids because they exhibit greater absorption in vivo compared to protein in monomeric form. Several factors are crucial for peptide absorption, such as the length of the peptides and their degradation by intestinal proteases. Generally, dipeptides and tripeptides cross intestinal membranes intact via peptide transport systems, while oligopeptides have a lower capacity for transport across the membranes compared to smaller peptides [41]. 

Based on their amino acid composition, size, sequence, and physicochemical properties, bioactive peptides offer numerous therapeutic alternatives for the prevention and treatment of various diseases and their comorbidities, including obesity [39,40]. 

The peptides in the studies covered in this SR came from bovine alpha-lactalbumin, cocoa seed (*Theobroma cacao* L.), chia seed (*Salvia hispanica* L.), rice bran (*Oryza sativa*), sesame (*Sesamum indicum* L.), sea buckthorn seed flour (*Hippophae rhamnoides*), and azuki bean (*Vigna angularis*) (Table 3). All characteristics of the peptides found, such as molecular mass, hydrophobicity, toxicity, stability, and isoelectric point, were also recorded (Table S2).

It is evident that there exists considerable variability among the characteristics of peptides, and their evaluation in studies is not uniform (Table S2). For instance, concerning molecular mass, the largest peptide evaluated in the studies was derived from adzuki beans, with a mass of 1389.62 Da, while the smallest peptide, derived from rice bran, had mass of 104.15 Da (Table S2). The peptides comprised sequences of 12 and 8 amino acids, respectively.

Nine dipeptides and twelve tripeptides from various sources were identified, while all the others had sequences containing more than 4 amino acids (aa), with the longest sequence comprising 13 aa [30,31,32,33,34,35,36] (Table 3). Regarding the toxicity profile of these peptides, among the 7 studies, 4 conducted evaluations and observed that the peptides derived from cocoa seeds, rice bran, sesame, and adzuki beans were non-toxic [31,33,34,36] (Table S2).

The predominant methods for peptide identification include protein extraction, hydrolysis, purification, identification, and the synthesis of peptide sequences, followed by the confirmation of bioactivity through in vitro and/or in vivo assays [42]. This entire process is both labor-intensive and costly. Consequently, several integrated bioinformatics techniques have been employed to predict the potential of these proteins for generating peptides, along with their evaluation on specific targets. Among the peptides covered in this systematic review, five were experimentally determined by mass spectrometry (MS): bovine alpha-lactalbumin, rice bran, sesame seed, sea buckthorn flour, and azuki bean [30,33,34,35,36]. In the studies evaluating peptides from chia seeds and cocoa beans, the molecules were designed using Marvin Sketch and Peptide Cutter software, respectively [31,32]. In silico analyses offer a more cost-effective means of screening and predicting the functionalities and characteristics of these peptides [42,43,44].

In silico studies with molecular docking are valuable tools for assessing peptide interactions with targets, which can be enzymes and receptors [39]. Typically, peptide–protein molecular docking studies involve the following steps: 1. Retrieval of the crystal structure of the targets/receptors from the RCSB Protein Data Bank (PDB) (http://www.rcsb.org/pdb/home/home.do/); 2. Generation or acquisition of the peptide structure using programs such as UniProt (https://www.uniprot.org/), Peptide Cutter (https://web.expasy.org/peptide_cutter/), ChemBio3D (https://biochemia.uwm.edu.pl/), and Hyperchem (http://www.hypercubeusa.com/), and obtaining the PDB (https://www.rcsb.org/) file; 3. Docking of the ligand with the receptor using different software packages such as GOLD (https://www.ccdc.cam.ac.uk/solutions/software/gold/), Autodock (https://autodock.scripps.edu/), HADDOCK (https://rascar.science.uu.nl/haddock2.4/), MOE (https://www.chemcomp.com/), among others; 4. Evaluation of interactions between peptide and target using different visualization programs such as Lig Plot (http://www.ebi.ac.uk/) and Studio Discovery (https://discover.3ds.com/) [39]. After these steps, the theoretical peptide models can be validated using servers such as MolProbity (http://molprobity.biochem.duke.edu/), along with analyses of bond sizes, angles, and Ramachandran plots to refine the theoretical structure obtained [45,46]. Following validation, these structures can be included in databases of proteins and biologically active peptides sequences, such as BIOPEP (https://biochemia.uwm.edu.pl/biopep-uwm/), which currently contains 5014 bioactive peptides [47].

All studies evaluated in this SR adhered to the outlined steps for conducting molecular docking studies; however, none conducted molecular dynamics simulations. Molecular dynamics, distinct from docking, yields insights into the dynamic behavior of a system, including atomic positions and velocities. It relies on temporal flexibility, enabling the investigation of conformational variations and deviations between two structures [48,49].

Molecular docking studies facilitate the identification of amino acid interactions between ligands and their respective targets, thereby aiding the scientific community to understand the mechanisms of peptide activity and in designing new peptides or enhancing the bioactivity of existing peptide structures [39]. Therefore, it is an essential tool for prospecting medications with functionality in various health conditions, such as obesity. In the treatment of obesity and its associated conditions—such as inflammation, hepatic fat accumulation, and insulin resistance—some synthetic peptides have already been tested in recent years [50,51,52]. 

Additionally, several peptides naturally produced by the human body have been extensively studied for their role in treating obesity. These peptides function as insulinotropic, regulate energy intake, and influence lipid metabolism and inflammation. Examples include glucagon-like peptide 1, leptin, neuropeptide Y, and adiponectin [53,54,55,56].

**Figure 3:**
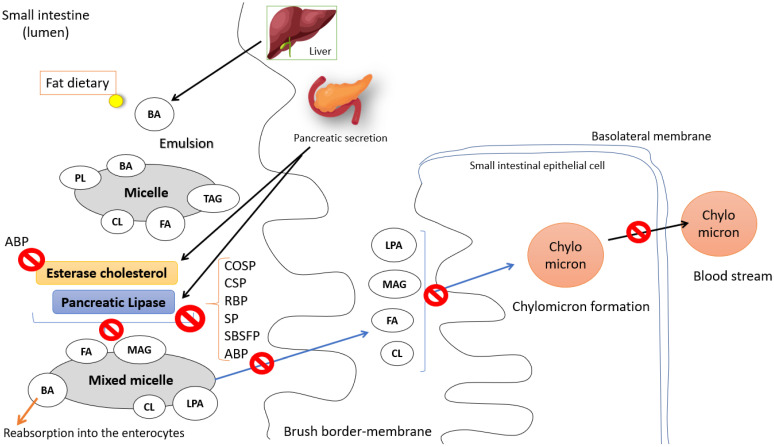
Possible mechanism of action of pancreatic lipase and cholesterol esterase inhibitory peptides in the digestion and absorption of fats. BA = bile acid; TAG = triglyceride; CL = cholesterol; FA = fatty acid; PL = phospholipid; LPA = lysophosphatidic acid; MAG = monoacylglycerol; COSP = cocoa seed peptides; CSP = chia seed peptides; RBP = rice bran peptides; SP = sesame peptides; SBSFP = sea buckthorn seed flour peptides; ABP = adzuki bean peptides; ⦸ indicates inhibition.

**Figure 4:**
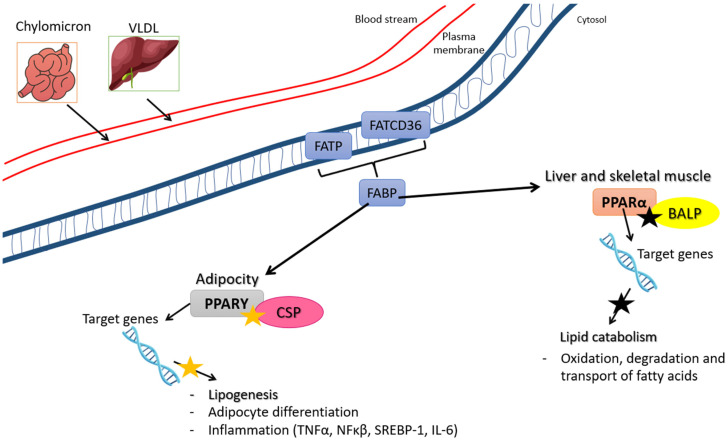
Possible mechanism of action of PPARγ and PPARα agonist peptides on lipid metabolism. PPARα = peroxisome proliferator-activated receptor alpha; PPARγ = peroxisome proliferator-activated receptor gamma; VLDL = very low-density lipoprotein; FATCD36 = fatty acid translocase; FATP = fatty acid transport protein; FABP = fatty acid binding protein; CSP = chia seed peptides; BALP = bovine alpha-lactalbumin peptides; TNFα = tumor necrosis factor alpha; NFκβ = nuclear factor kappa beta; SREBP-1 = sterol regulatory element binding protein 1; IL-6 = interleukin 6; black star indicates activation; orange star indicates blocking.

The peptides investigated in the studies included in this systematic review were tested against various in silico targets related to obesity, including peroxisome proliferator-activated receptor alpha (PPARα), pancreatic lipase (PL), peroxisome proliferator-activated receptor gamma (PPARγ), cholesterol esterase (CE), fatty acid synthase (FAS), and monoacylglycerol lipase (MAGL). Of these, the first four were re-evaluated in in vitro and/or in vivo experiments. Regarding the potential molecular mechanism of action observed (Figure 3 and Figure 4), all peptides evaluated in the studies demonstrated some form of interaction with the target protein in the molecular docking analyses, exhibiting favorable theoretical affinity.

#### 3.3.2. Anti-Obesity Peptides: Pancreatic Lipase (PL) and Cholesterol Esterase (CE) Inhibitors

Among the seven studies, five suggested that at least one modeled peptide could serve as a potential inhibitor of lipase and cholesterol esterase in silico. These studies also evaluated the inhibitor type of these peptides through in silico and enzyme kinetic analyses using Lineweaver–Burk plots [31,33,34,35,36] (Table S2).

In the studies reviewed in this SR, five examined pancreatic lipase (PL) as a possible target for their peptides. PL is extensively studied and holds significance in the context of obesity prevention and treatment, as it is the primary enzyme responsible for digesting dietary fats. Produced by the acinar cells of the pancreas, PL hydrolyzes 50 to 70% of triglycerides (TG) in the diet [57]. PL inhibition would reduce TG hydrolysis, slowing the absorption of fatty acids into systemic circulation and in adipocytes [58].

Currently, orlistat (tetrahydrolipstatin) is the only PL inhibitor approved by the FDA, and its long-term use is recommended to mitigate the absorption of dietary lipids [58]. Nevertheless, orlistat is associated with numerous side effects, including allergic reactions, headaches, dizziness, dry mouth with a bitter taste, oily bowel movements, rectal pain, as well as swelling of the face, throat, and tongue [31,59].

The benefits of utilizing PL inhibitors stem from their inability to penetrate human blood vessels or the nervous system. In addition, they do not disrupt the water–electrolyte balance or bone circulation, making PL inhibitors relatively safe [59]. Among the studies encompassed this SR, four evaluated the toxicity of their lipase inhibitor peptides and observed that they did not present toxicity (Table S2).

Given the adverse reactions related to the long-term use of orlistat, plant-derived PL inhibitors, which have demonstrated significant bioactive potential as anti-obesity agents, have been the focus of numerous studies [58,59]. This SR examined the effects of such peptides as possible PL inhibitors [31,33,34,35,36].

Molecular docking analyses are based on the binding affinity between a potential ligand, obtained from a prototype, and a molecular target (receptor, enzyme). This method can predict interactions and binding energy and characterize binding modes [60,61,62].

The peptide FYLGYCDY, derived from rice bran, demonstrated the ability to inhibit porcine pancreatic lipase (PPL), and according to the Lineweaver–Burk plots, it is a non-competitive inhibitor. Non-competitive inhibitors bind to a site different from the enzyme’s catalytic site, thus binding to an allosteric site. This binding induces conformational changes in its structure, altering the conformation of the catalytic site and hindering effective substrate interaction, consequently impeding product formation [63]. 

However, molecular docking analysis, aimed at predicting the binding site of the FYLGYCDY peptide with the PPL complex, revealed a competitive binding pattern. It binds to the Phe216 binding site with a high docking score (122.54) (Table S3). Competitive inhibitors bind to the enzyme’s active site, obstructing substrate binding and preventing product formation [63].

In molecular docking, a higher docking score and lower binding energy indicate a greater binding affinity and higher potential of the ligand tested in silico [60,61,62]. Accordingly, the rice-bran-derived peptide demonstrated the ability to competitively inhibit lipase in silico with high theoretical affinity.

Three peptides (NIF, QWM, and TF) derived from sesame exhibited non-competitive inhibition, while two (EW and AGY) demonstrated mixed-type inhibition against human pancreatic lipase (HPL) by the Lineweaver–Burk method (Table S2). In mixed-type inhibition, the inhibitor binds to a region of the enzyme distinct from the active site but does not interfere with substrate binding to the catalytic site. This type of inhibitor can bind to both the free enzyme and the enzyme-substrate complex, forming an enzyme–substrate–inhibitor complex, which is inactive and prevents catalysis [63]. In the docking analysis, peptides derived from sesame directly bound to Ser152 and His263 in the catalytic triad of PPL and exhibited high theoretical affinity (−7.4 to −8.1 kcal/mol) for lipase [34] (Table S2). 

The evaluation of the binding site on the target is crucial for assessing the affinity, shape, and inhibition capacity of these peptides [60,61,62]. Peptides derived from sesame not only inhibited lipase with high affinity but also bound to its catalytic site, likely significantly reducing the enzyme’s activity [34].

Sea buckthorn peptides are non-competitive against PPL, as determined by Lineweaver–Burk method evaluation (Table S2). In the in silico analysis, all of these peptides displayed hydrophobic interactions between their aa and hydrophobic residues of PLL, along with high theoretical affinity for PPL. Specifically, VR (−5.2 kcal/mol), EEAASLR (−5.5 kcal/mol), RDR (−5.4 kcal/mol), and NLLHR (−4.8 kcal/mol) demonstrated these characteristics. Additionally, the VR dipeptide had at least one type of interaction with PPL [35].

Studies in the existing literature have already investigated the enzymatic kinetics of inhibitory peptides. For instance, in 2018, Medeiros et al. [64] reported that a trypsin inhibitor derived from tamarind seeds exhibited competitive inhibition according to the Lineweaver–Burk method and molecular docking analyses. Similarly, another study in the same year observed that hazelnut-derived peptides were non-competitive for angiotensin-converting enzyme (ACE) [59]. Additionally, a study observed non-competitive inhibition in the case of wheat α-amylase inhibitor [65].

Several studies have evaluated the enzymatic kinetics of peptides derived from natural sources against various enzymes for diverse applications, showing different modes of inhibition. However, in silico studies utilizing molecular docking techniques can analyze the interaction between peptides and their target amino acids, elucidating the ligand–target relationships and possible modes of inhibition [39].

Regarding the possible molecular mechanism, two studies evaluated their peptides as prospective inhibitors through in silico analyses in this SR without evaluating the enzyme kinetics using the Lineweaver–Burk method [30,31]. Among the peptides derived from cocoa, four showed the highest theoretical affinity for human pancreatic lipase (HPL) in the in silico analysis: EEQR (−6.5 kcal/mol), GGER (−6.3 kcal/mol), QTGVQ (−6.2 kcal/mol), and VSTDVNIE (−6.1 kcal/mol) (Table 4 and Table S3) [31]. Furthermore, the tetrapeptide EEQR demonstrated binding to HPL at 9 aa residues (Lys239, Arg265, Tre271, Asp88, Tyr267, Asn92, Ser333, Asp331, and Lys268) with different types of interactions (Table 4 and Table S3). Thus, cocoa-derived peptides were demonstrated to be potent lipase inhibitors in silico, exhibiting diverse peptide–ligand interactions [31].

The study that evaluated peptides derived from adzuki beans not only observed PL inhibition but also identified, through in silico studies, the inhibition of cholesterol esterase (CE) (Table 4). CE is a nonspecific enzyme that can hydrolyze a broad spectrum of substrates, namely cholesterol esters, TG, and certain phospholipids. Its function goes beyond dietary lipid digestion; it also plays a crucial role in transporting free cholesterol from micelles to enterocytes, a process closely linked to obesity and hypercholesterolemia [66].

This study suggested that, in addition to PPL, peptides from adzuki beans also acted as inhibitors of CE, with docking interactions evaluated for both enzymes [36]. The five peptides assessed interacted with 7 to 11 aa residues of PPL and 7 to 16 aa residues of CE, exhibiting high theoretical affinity for PPL (from −116.5 to −126.7 kcal/mol) and CE (from −132.1 to −148.9 kcal/mol) (Table 4 and Table S3). Among these peptides, three (LGLDSSLLPH, FDTGSSFYNKPAG, and IFNNDPNNHP) bind to catalytic residues of PPL (Ser153 and His264) and other residues (Ala261, Phe259, and Val260). Four peptides (LLGGLDSSLLPH, FDTGSSFYNKPAG, IWVGGSGMDM, and YLQGFGKNIL) occupy the catalytic site of CE (Ser194 and His435), while LLGGLDSSLLPH, FDTGSSFYNKPAG, IWVGGSGMDM, and IFNNDPNNHP inhibit CE activity by binding to the substrate binding sites (Ala108) (Table 4 and Table S3). Azuki bean peptides inhibit PPL and CE by binding to the catalytic site of the enzymes with high theoretical affinity, demonstrating significant inhibition power [36].

Some CE inhibitors have been previously reported; the most recent was based on phosphorylated flavonoids [67]. However, the inhibition of CE with peptides of natural origin has not yet been documented. Therefore, the development of effective CE inhibitors with high specificity becomes an endeavor to explore peptides and their anti-obesity effects.

All studies evaluating the inhibition of PL and CE suggest that inhibiting enzyme activity may prevent the formation of mixed micelles and their intestinal absorption at the brush border membrane. Consequently, this inhibition could reduce the formation of chylomicrons and fat absorption, leading to a decrease in fat accumulation (Figure 3) [68,69,70].

#### 3.3.3. Anti-Obesity Peptides: Peroxisome Proliferator-Activated Receptor Alpha Type (PPARα) and Peroxisome Proliferator-Activated Receptor Gamma Type (PPARγ) Agonists

PPARs belong to a class of nuclear receptors that respond to endogenous ligands, including fatty acids (FAs) and their metabolites, with prominent members being PPARγ and PPARα. Upon binding to their respective ligands, PPARs form heterodimers with retinoid X receptors and bind to a specific DNA response element (PPRE) to regulate transcription and gene expression [71,72]. PPARγ is predominantly expressed in adipose tissue, being a regulator of lipid metabolism and the adipogenesis process, while PPARα is an important factor in lipid catabolism [71,72]. 

The other two studies suggested, through in silico analyses, that their peptides could act as agonists (Table 4). Peptides derived from bovine alpha-lactalbumin are potential agonists of the peroxisome proliferator-activated receptor alpha type (PPARα), while peptides from chia seed are agonists of the peroxisome proliferator-activated receptor gamma type (PPARγ), fatty acid synthase (FAS), and monoacylglycerol lipase (MAGL) [30].

In the study evaluating four peptides derived from alpha-lactalbumin, all of them exhibited binding to Met355 in the active site of PPARα through hydrophobic interactions [30]. Particularly, the tripeptide named P8 formed hydrophobic interactions with four other residues of PPARα (His440, Tyr464, Tyr314, and Ser280) and demonstrated a lower binding free energy (ELL) of −7.86 kcal/mol, indicating higher theoretical affinity [30].

Regarding the investigation into peptides derived from chia seeds, the two peptides showed interactions with several amino acids of the enzymes FAS, MAGL, and PPARγ receptor [32]. Pep2 showed the highest theoretical affinity for PPARγ with an ELL of −6.9 kcal/mol and for MAGL with an ELL of −7.3 kcal/mol. On the other hand, Pep1 demonstrated the greatest interaction with FAS, with an ELL of −7.3 kcal/mol [32].

Studies identifying peptides as PPARα and PPARγ agonists suggest that the anti-obesity mechanism is likely due to the modulation of key factors involved in lipid metabolism, adipogenesis, and inflammation [30,32] (Figure 4).

These findings underscore the importance of utilizing bioinformatics tools for screening and evaluating potential effects on the organism, given cost-effectiveness and accessibility. Such tools can be employed to identify the specificity, affinity, and interaction of ligands, such as peptides, with their potential targets [73]. However, the therapeutic potential of these peptides/ligands can only be realized and studied with greater precision through in vitro and/or in vivo experimental testing.

#### 3.3.4. Reassessment of In Silico Studies Using In Vitro Models

In this SR, all studies re-evaluated the peptides examined for characteristics observed during docking in in vitro and/or in vivo studies. Six of seven studies tested the in vitro pancreatic lipase (PL) inhibition of its peptides [31,32,33,34,35,36] (Figure 3; Table 4). It is important to emphasize that the studies are not homogeneous regarding the type of lipase used, with human [36], bovine [32], and predominantly (three studies) porcine lipases [31,33,34,35] employed, with porcine lipase being the most studied commercial lipase [74]. A limitation in comparing these in vitro results arises from the use of different types of lipases, which complicates comparisons [74]. Additionally, the careful selection of structures used in silico is necessary to ensure reproducibility in computational evaluations [14,15,16,17].

Irrespective of the type of lipase, the results showed that in all six studies, peptides derived from cocoa seed, chia, rice bran, sesame, sea buckthorn, and adzuki beans were able to inhibit lipase in vitro, corroborating the findings from molecular docking [31,33,34,35] (Figure 3). In silico analysis showed that five of these studies assessed the interaction of their peptides with pancreatic lipase [33,34,35], with two employing human lipase [31,34] and three utilizing porcine lipases [33,35,36] (excluding the chia-derived peptides, which targeted other receptors in docking [32]), yielding promising theoretical affinity and interactions with various PL amino acid residues [31,33,34,35,36] (Table 4).

In addition to being tested for PL inhibition under normal conditions, sea buckthorn peptides were also evaluated for inhibitory rates with increased temperature, and after simulated gastrointestinal digestion (DGIS), good stability of PL inhibition by the peptides was obtained even with these conditions [35]. Inhibiting PL and reducing lipid absorption is a promising approach for discovering anti-obesity agents [59].

The study evaluating peptides derived from chia seeds encompassed not only PL inhibition but also in vitro assessments using 3T3-L1 adipocytes [32]. These evaluations included examining cell viability, the effects of peptides on the differentiation process, cellular lipid accumulation measured by Oil Red O staining, and the expression level of proteins associated with adipogenesis and inflammatory pathways (e.g., PPARγ, TNFα, NFκβ, SREBP-1, LPL, IL-6, and 10), as well as TG content [32] (Figure 4; Table 4). The results indicate not only the inhibition of lipase activity but also increased cell viability, reduced lipid and TG levels, and diminished expression of PPARγ, TNFα (with Pep2), nuclear factor kappa beta (NFκβ), lipoprotein lipase (LPL), sterol regulatory element binding protein 1 (SREBP-1) (with Pep2), IL-6, and IL-10 [32] (Figure 4; Table 4).

In silico, the authors investigated three targets involved in lipid metabolism and adipogenesis (PPARγ, FAS, and MAGL) [32]. PPARγ is recognized for its role in both processes associated with obesity, and it is also implicated in the negative regulation of NFκβ, thereby affecting not only NFκβ but also pro-inflammatory signaling molecules such as IL-6, which are linked to inflammation in obesity [71,72]. The presence of chia peptides resulted in a decrease in the expression of these two markers.

Some authors have posited that the modulation and prevention of obesity might involve the regulation of genes associated with adipogenesis such as PPARγ, SREBP-1, FAS, and LPL, among others [75,76]. Consistent with the findings from the in vitro reassessment, chia peptides reduced the protein expression of PPARγ, SREBP-1, and LPL [32]. Furthermore, molecular docking studies indicated that chia Pep2 had higher theoretical affinity for PPARγ (−6.9 kcal/mol) and MAGL (−7.3 kcal/mol), while Pep1 displayed greater affinity for FAS (−7.3 kcal/mol), suggesting a potential target and corroborating the observed in vitro effects [32] (Table 4).

The investigation examining peptides derived from bovine alpha-lactalbumin involved conducting in vitro experiments using HepG2 cells [30]. These experiments included assessments of cell viability, Oil Red O staining to measure FFA accumulation and TG levels, an assay for intracellular PPARα content, quantitative real-time PCR (qRT-PCR) for PPARα gene expression, and Western blot analysis for PPARα protein level (Table 4) [30]. It is important to highlight that PPARα is essential for lipid catabolism, being a key factor in the evaluation of anti-obesity peptides [71,72].

The peptides derived from bovine alpha-lactalbumin demonstrated in silico binding to the active site of PPARα with favorable theoretical affinity. In vitro experiments revealed increased cell viability, reduced levels of FFA and TG, as well as enhanced gene and protein expression of PPARα in HepG2 cells (Figure 4). These findings confirm the potential of alpha-lactalbumin peptides as PPARα agonists, capable of modulating lipid metabolism and adipogenesis by reducing FFA and TG content.

The study assessing adzuki bean peptides, in addition to PL inhibition, also tested CE inhibition. It was observed that the peptides effectively inhibited both PL and CE in vitro, consistent with the predictions from docking studies, wherein the peptides had high theoretical affinity for the enzymes and bound to their active and substrate binding sites (Figure 3). It is well-established that CE is closely linked to obesity and hypercholesterolemia by regulating lipid metabolism [44], as does PL [59].

In vitro research methods in drug screening and discovery offer a cost-effective platform for identifying novel anti-obesity drugs [77]. These studies present several advantages including greater control over experimental variables such as simulating specific conditions in cells (e.g., inflammation), reduced use of laboratory animals, and the ability to evaluate specific cell lines, even genetic manipulation [77,78].

Therefore, in assessing factors related to obesity, such as lipid metabolism, adipogenesis, and inflammation, the in vitro tests may be an opportunity for personalized therapy [77]. They are readily accessible, rapid, and enable high-throughput screening. In vitro studies yield crucial data on drug–target interactions, mechanisms of action, and toxicity profiles of the drugs [77,78,79].

However, while in vitro studies provide valuable findings, they are limited in their ability to fully replicate the complexities of a biological system [79]. Consequently, in vivo studies have emerged to address this challenge and validate the findings obtained in vitro [79].

#### 3.3.5. Reassessment of In Silico Studies Using In Vivo Models

The investigation that evaluated peptides derived from cocoa seeds was the sole study to conduct not only in vitro PL inhibition testing but also re-evaluation of the peptides in an animal model [31]. The studies utilized male Sprague Dawley rats fed a high-fat diet (HF) and treated with cocoa seed peptides (Table 4). The peptides derived from the cocoa seed showed high theoretical affinity and bound to different aa residues of the PL. In vitro experiments demonstrated the inhibition of PL activity. In vivo, the administration of these peptides to animals fed an HF diet resulted in decreased total lipid content and fecal TG, as well as a reduction in fat absorption rates, despite not significantly affecting body weight or fecal total cholesterol (TC), corroborating the data evaluated in docking and showing lipase inhibition in these animals [31] (Table 4).

Animal models offer distinct advantages over in vitro models, as they can more accurately represent human representation, particularly in the context of managing specific diets and mimicking the obesogenic environment [79]. By closely aligning with the biological system, animal models provide a more promising means to evaluate the management of obesity and its effects. A limitation of animal models is the lack of standardization across studies, including variations in the types of diets employed [80]. Integrating both in vitro and in vivo studies can provide more robust and reliable findings, ensuring the applicability of results in future clinical investigations.

### 3.4. Limitations of the Study

One of the limitations of the studies comprising this SR is their heterogeneity regarding peptides characteristics. These peptides varied in sizes, sequences, methods of acquisition, and hydrophobicity, among other factors. Another limitation of these studies is the selection of in silico targets, with three studies using porcine target structures; however, a positive point related to the structure was that all studies were classified as having a low risk of bias regarding the method of obtaining the structure, that is, all 3D structures of the targets came from the Protein Data Bank (PDB), with none derived theoretically.

Within the context of this SR aimed at assessing the efficacy of the in silico approach as a screening method for anti-obesity peptides with subsequent vitro/in vivo validation, a notable consideration arises. Some in silico studies evaluated specific peptides that demonstrated interactions with the anti-obesity target. However, upon re-evaluation in in vitro/in vivo studies, the majority (four) of these studies did not use pure peptides but instead utilized protein hydrolysates. This practice complicates to elucidation the true action and mechanism of the peptides underlying the observed results.

Bioinformatics through in silico studies has emerged as a prominent method for drug screening across a spectrum of conditions, including obesity. These studies provide crucial information about the interaction between ligand and targets, as well as potential biological effects. Therefore, within this SR, only studies were included that underwent some form of reassessment, either through computational tools, in vitro experimentation, and/or in vivo models. This rigorous approach aims to ensure the applicability and safety of these peptides as new therapeutic alternatives for obesity.

## 4. Conclusions

The present study represents a promising approach to exploring new therapeutic options for obesity using natural bioactive peptides, integrating bioinformatics analyses with in vitro and/or in vivo experimental models. Although in silico studies hold significant untapped potential, there are still relatively few studies that correlate in vitro and in vivo findings with in silico data, which could help identify shortcomings and improve the techniques used in molecular modeling and other parameters employed by software. Another limitation is the need to define the critical variables for each process studied, as these factors can directly influence the results obtained. In this systematic review, all peptides evaluated through molecular docking demonstrated in vitro and/or in vivo findings consistent with predictions generated by computational simulation. Therefore, the combination of bioinformatics investigations with assessments in experimental models unveils possibilities in research into obesity therapy. In silico screening studies enable time and cost savings, as the molecule and its viability are first assessed in silico, and then more assertive in vitro and in vivo studies can be developed. In summary, there is an importance of cost-effectiveness in the process of evaluating anti-obesity peptides.

## Figures and Tables

**Table 1:** Elements of the research question, based on the problem, exposure, and context (PECo) framework, to address the question: “Which peptides or proteins have been used to treat obesity in the in silico studies?”.

Abbreviation	Descriptor	Elements of the Question
**P:**	Problem	Peptides and/or proteins used in the treatment of obesity
**E:**	Exposure	Obesity
**Co:**	Context	In silico studies

PECo (P, problem; E, exposure; Co, context).

**Table 2:** Search strategies for each database to be retrieved to answer the SR question: “Which peptides or proteins have been used to treat obesity in the in silico studies?”.

Database	Search Equation
**PUBMED** Selected filter: Title/Abstract	(((protein[Title/Abstract] OR peptide[Title/Abstract] OR treatment[Title/Abstract]) AND (in silico[Title/Abstract] OR computer simulation[Title/Abstract])) AND (molecular dynamic simulation[Title/Abstract] OR molecular dynamic[Title/Abstract] OR molecular docking simulation[Title/Abstract] OR molecular docking[Title/Abstract]) AND (Obesity[Title/Abstract] OR Obese[Title/Abstract]))
**SCOPUS** Selected filter: Article title, abstract, keywords	TITLE-ABS-KEY (“protein” OR peptide OR treatment) AND TITLE-ABS-KEY (“in siilico” OR “computer simulation”) AND TITLE-ABS-KEY (“molecular dynamic simulation” OR “molecular dynamic” OR “molecular docking simulation” OR “molecular docking”) AND TITLE-ABS-KEY (“obesity” OR “obese”)
**SCIENCE DIRECT** Selected filter: Title, abstract or author-specified keywords	(Protein OR peptide) AND (in silico OR computer simulation OR molecular dynamic simulation OR molecular dynamic OR molecular docking simulation OR molecular docking) AND (obesity)
**EMBASE** Selected filter: Title, abstract or author keywords	(protein*:ti,ab,kw OR peptide*:ti,ab,kw) AND (‘in silico*’:ti,ab,kw OR ‘computer simulation*’:ti,ab,kw) AND (‘molecular dynamic* simulation*’:ti,ab,kw OR ‘molecular dynamic*’:ti,ab,kw OR ‘molecular docking* simulation*’:ti,ab,kw OR ‘molecular docking*’:ti,ab,kw) AND (obesity*:ti,ab,kw OR obese*:ti,ab,kw)
**VIRTUAL HEALTH LIBRARY** Selected filter: Title, abstract, subject	(protein* OR peptide* OR treatment*) AND (in silico* OR computer simulation*) AND (molecular dynamic* simulation* OR molecular dynamic* OR molecular docking* simulation* OR molecular docking*) AND (obesity* OR obese*)
**WEB OF SCIENCE**	(((ALL = (protein* OR peptide*)) AND ALL = (in silico* OR computer simulation*)) AND ALL = (molecular dynamic* simulation* OR molecular dynamic* OR molecular docking* simulation* OR molecular docking*)) AND ALL = (Obesity* OR Obese*)

**Table 3:** Characteristics of studies and peptides evaluated in the SR.

Authors/Year	Peptides/ Sequence *	Origin	Original Protein	Software for Obtaining Proteins/Projecting Peptides
Chen et al. (2021) [30]	P2 (GINY) P8 (DQW) P13 (DQWL) P14 (LFQ)	Bovine alpha-lactalbumin	Alpha-lactalbumin fraction 3	UniProt (https://www.uniprot.org/)
Coronado-Cáceres et al. (2020) [31]	EEQR GGER TIAV AGRP VTDG NTQR EQCQR VTDG NQGAI QTGVQ VSTDVNIE HSDDDGQIR SDNE CSTSTV	Cocoa beans (*Theobroma cacao* L.)	**Vincilin:** EEQR GGER TIAV AGRP VTDG NTQR EQCQR VTDG NQGAI **Albumin:** QTGVQ VSTDVNIE HSDDDGQIR SDNE CSTSTV	UniProt (https://www.uniprot.org/) PeptideCutter (https://web.expasy.org/peptide_cutter/) MarvinSketch (ChemAxon Ltd., Boston, MA, EUA) (https://chemaxon.com/marvin)
Grancieri et al. (2021) [32]	NSPGPHDVALDQ (PEP1) RMVLPEYELLYE (PEP2)	Chia seed (*Salvia hispanica* L.)	Glutelin fraction	MarvinSketch (ChemAxon Ltd., Boston, MA, EUA) (https://chemaxon.com/marvin)
Ketprayoon et al. (2021) [33]	FYLGYCDY	Rice bran (*Oryza sativa*) defatted (DORB)	DORB Fraction 5 through the use of Alcalase^®^	Discovery Studio 2019 (https://www.3ds.com/products/biovia/discovery-studio)
Wang et al. (2022) [34]	EW NIF AGY PIF QWM TF	Sesame (*Sesamum indicum* L.)	11S globulin and 2S albumin	UniProt (https://www.uniprot.org/) ExPASy PeptideCutter (https://web.expasy.org/peptide_cutter/)
Xiang et al. (2020) [35]	LR VR APYR DR EEAASLR ELR EWR FLR FMDR FR ALR LLR MR NLLHR PECR PR QR RDR SDR TR WR WRN	Sea buckthorn seed flour (*Hippophae rhamnoides*)	Hawthorn seed hydrolyzate identified by HPLC/MS/MS	ChemBio3D (https://biochemia.uwm.edu.pl/biopep-uwm/)
Zhao et al. (2024) [36]	LLGGLDSSLLPH FDTGSSFYNKPAG IWVGGSGMDM YLQGFGKNIL IFNNDPNNHP	Adzuki beans (*Vigna angularis*)	Fraction 1 (<3 kDa) of adzuki bean protein hydrolysate	UniProt (https://www.uniprot.org/)

* A: alanine; C: cysteine; D: aspartic acid; E: glutamic acid; F: phenylalanine; G: glycine; H: histidine; I: isoleucine; K: lysine; L: leucine; M: methionine; N: asparagine; P: proline; Q: glutamine; R: arginine; S: serine; T: threonine; V: valine; Y: tyrosine; W: tryptophan.

**Table 4:** Characteristics and main results of in silico studies with in vitro/in vivo re-evaluation.

			IN SILICO STUDIES		IN VITRO/IN VIVO REASSESSMENT		
Reference	Methodology	Origin of Peptides	In Silico Target	Main Residues in the Interaction Interface of the Most Promising Agent/Main Results of Docking or Molecular Dynamics	Technique/Types of Culture/Strain/Diet	Treatment/Main Effects	Possible Molecular Mechanism of Application
Chen et al. (2021) [30]	Docking Software AutoDock Vina (https://vina.scripps.edu/)	Bovine alpha-lactalbumin	PPARα	Met355 P8 (His440, Tyr464, Tyr314, and Ser280) and highest theoretical affinity (−7.86 kcal/mol)	1. Cell viability 2. Oil Red O staining and TG levels 3. Intracellular content assay 4. Gene expression by quantitative real-time PCR (qRT-PCR) (Effects of P2 and P8) 5. Western blot Cell model: HepG2 cells	↑ Cellular viability ↓ FFA and TG content ↑ PPARα gene and protein expression	Agonist
Coronado-Cáceres et al. (2020) * [31]	Docking Software AutoDock Vina (https://vina.scripps.edu/)	Cocoa beans (*Theobroma cacao* L.)	HPL	Highest theoretical affinity: EEQR (−6.5 kcal/mol), GGER (−6.3 kcal/mol), QTGVQ (−6.2 kcal/mol), and VSTDVNIE (−6.1 kcal/mol) EEQR at Lys239, Arg265, Tre271, Asp88, Tyr267, Asn92, Ser333, Asp331, and Lys268	1. Pancreatic Lipase Inhibition (PPL) 2. Male Sprague Dawley Rats Fed HF Diet	× PPL ↑ Total fecal lipids and fecal TG ↓ Fat absorption rate Ø Body weight and fecal TC	Inhibitor
Grancieri et al. (2021) [32]	Docking Software AutoDock Vina (https://vina.scripps.edu/)	Chia seed (*Salvia hispanica* L.)	PPARγ FAS MAGL	**PPARγ** PEP1 = Lys230; Ala235; Lys232; Ala231; Tyr219; Glu378; Arg234 PEP2 = GLN420; TYR219; LYS224; ILE223; LYS232; THR241; ALA231; ARG234; GLU378; ASP380; HIS425 **MAGL** PEP1 = ARG98; ASP26; SER91; VAL90; VAL95; VAL78; CYS208; ILE211; SER218; LYS160 PEP2 = MET123; SER122; SER155; LEU148; LEU213; ALA151; ALA156; LEU214; LEU150; PRO153; SER218; ARG222; LYS160; ALA164; ALA163; ILE211; LEU167; CYS208; VAL207 **FAS** PEP1 = GLU2227; GLY2228; TYR2288; PRO2229; CYS2292; LYS2436; ASP2291; THR2434; ARG2275; ARG2421; ARG2428; TYR2433; ILE2282; HIS2283; SER2281; LEU2279; ASP2280 PEP2 = SER2281; GLU2227; LYS2436; TYR2288; THR2230; PRO2229; GLN2432 PEP2 = greater interaction with PPARγ with ELL (−6.9 kcal/mol) and with MAGL with ELL (−7.3 kcal/mol) PEP1 = greater interaction with FAS (−7.3 kcal/mol)	1. Cell viability 2. Inhibition of BPL 3. Effects of peptides on 3T3-L1 adipocytes during the differentiation process 4. Effect of peptides on cellular lipid accumulation by Oil Red O 5. Influence of peptides on the expression of proteins related to adipogenesis and inflammatory processes (Western Blot) 6. Effects of peptides on TG content	↑ Cellular viability × LPB ↓ FFA and TG content ↓ PPARγ ↓ TNFα (Pep2) ↓ NFκβ ↓ LPL ↓ SREBP-1 (Pep2) ↓ IL-6 and IL-10	Agonist
Ketprayoon et al. (2021) [33]	Docking Software GOLD 5.7.1 (https://www.ccdc.cam.ac.uk/solutions/software/gold/)	Rice bran (*Oryza sativa*) defatted (DORB)	PPL	Phe216, Ser153, Asp177, and His264 Docking score = 122.54	1. Pancreatic Lipase Inhibition (PPL)	× PPL	Inhibitor
Wang et al. (2022) [34]	Docking Software AutoDock Vina (https://vina.scripps.edu/)	Sesame (*Sesamum indicum* L.)	HPL	The 6 peptides can interact with Phe77, His151, Ser152, Phe215, and His263 They have high theoretical affinity (−7.4 to −8.1 kcal/mol)	1. Pancreatic Lipase Inhibition (PPL)	× PPL	Inhibitor
Xiang et al. (2020) [35]	Docking Software AutoDock Vina (https://vina.scripps.edu/)	Sea buckthorn seed flour (*Hippophae rhamnoides*)	PPL	EEAASLR (Val322, Gln324, and Gln188); NLLHR (His224 and Asn320); RDR (Ser323, Val322, and Gln 324) and VR (Pro194) VR had at least one type of interaction with LPP High theoretical affinity for LPP. VR (−5.2 kcal/mol); EEAASLR (−5.5 kcal/mol); RDR (−5.4 kcal/mol); NLLHR (−4.8 kcal/mol)	1. Determination of the inhibitory rate of SSPH against PPL 2. Determination of kinetics and mode of inhibition in PPL 3. Thermal stability of SSPH inhibitory activity against PPL SSPH 4. The inhibitory activity of SSPH against PPL before and after GIS	× PPL under normal conditions × PPL with temperature rise and after GIS	Inhibitor
Zhao et al. (2024) [36]	Docking Software Dock (https://dock.compbio.ucsf.edu/)	Adzuki beans (*Vigna angularis*)	PPL CE	PPL = LLGGLDSSLLPH, FDTGSSFYNKPAG and IFNNDPNNHP (Ser153, His264, Ala261, Phe259, and Val260) CE = LLGGLDSSLLPH, FDTGSSFYNKPAG, and IWVGGSGMDM (Ser194, His435, and Ala108) YLQGFGKNIL (Ser194 and His435) IFNNDPNNHP (Ala108) High theoretical affinity for PPL (from −116.5371 to 126.7088 kcal/mol) and CE (from −132.1017 to −148.9364 kcal/mol)	1. Pancreatic Lipase Inhibition (PL) ** 2. CE inhibition assay	× PL × CE	Inhibitor

* Only study that re-evaluated in vitro and in vivo experiments. ** Does not indicate the origin of the LP (human, porcine, or bovine). Legend: Amino acids: Asp—aspartic acid; Ala—alanine; Arg—arginine; Asn—asparagine; Cys—cysteine; Phe—phenylalanine; Gly—glycine; Gln—glutamine; His—histidine; Leu—leucine; Lys—lysine; Met—methionine; Ser—serine; Tyr—tyrosine; Thr—threonine; Trp—tryptophan; Val—valine. Symbols: ↑ increase; ↓ decrease; × inhibition; Ø there was no significant effect on the parameter. HF: high-fat diet; TC: total cholesterol; FFA: free fatty acids; TG: triglycerides; PPARα: peroxisome proliferator-activated receptor alpha type; HPL: human pancreatic lipase; BPL: bovine pancreatic lipase; PPL: porcine pancreatic lipase; PL: pancreatic lipase; FAS: fatty acid synthase; MAGL: monoacylglycerol lipase; PPARγ: peroxisome proliferator-activated receptor gamma type; TNFα: tumor necrosis factor alpha; NFκβ: nuclear factor kappa beta; LPL: lipoprotein lipase; SREBP-1: sterol regulatory element binding protein 1; IL-6: interleukin 6; IL-10: interleukin 10; GIS: gastrointestinal digestion simulation; CE: cholesterol esterase.

## Supplementary Materials

The supporting information can be downloaded at: https://www.mdpi.com/article/10.3390/ijms25179646/s1.
