# Supplementary material for: Peptides Evaluated In Silico, In Vitro, and In Vivo as Therapeutic Tools for Obesity: A Systematic Review
Source: Int J Mol Sci. 2024 Sep 6;25(17):9646. doi: 10.3390/ijms25179646 (PMC11395041; doi:10.3390/ijms25179646)
Supplement: Supplementary file 1 [file ijms-25-09646-s001.zip › ijms-3159018-supplementary.pdf]

Table S1. Form for assessing the risk of bias in in silico studies.

Bias Domain	issue	Low Risk of Bias	High Risk of Bias	Unclear Risk of Bias
**Ligand selection**	ligand filtering	Should be performed	Did not apagied	No date
**Ligands optimization**	Ionization assessment	The ligands were ionized according to pKa and pH values of media	The research was performed without reference to pKa values of ligands and pH values of media	No date
	Generation of energetically possible conformations	Should be performed	Generation was performed without reference to potential energy calculation	No date
**Target selection**	Resolution of protein structure	Not more than 2.5 Å	More than 2.5 Å	No date
	Method of protein target structure obtaining	NMR spectroscopy or Xray crystallography	Cryogenic electron microscopy or modeling	No date
**Target optimization**	Control of histidine protonation	Should be performed	The structure of target did not reference biological conditions	No date
	Protonation of amino acids after X-ray crystallography or cryogenic electron microscopy	Should be performed	The structure of target did not reference biological conditions	No date
	Addition of missing residues and side chains after X-ray crystallography or cryogenic electron microscopy	Should be performed	Was performed without special tools	No date
	Addition of metals	Should be performed	The structure of target did not reference biological conditions	No date
**Docking**	Molecular docking software	Glide, GOLD	AutoDock , DOCK, FlexX	No date
**Results assessment**	Visual control	Should be performed	Structure defects were observed	No date
	Re-docking	Should be performed	The RMSD value is too high compared with the initial structure	No data
	Verification of docking results by in vitro study in vitro or in vivo	Binding constant should be determined or performing in an in vitro or in vivo study.	The quantitative calculations were not performed	No data

Source: Adapted from Taldaev et al. by the author.

Table S2. Characteristics of the peptides found in the studies.

Authors/ Year	Peptides/Sequence*	Origin	Original protein	Software for obtaining proteins/projecting peptides	Molecular mass (Da)	Hydrophobicity	Solubility in water	Stability	Toxicity	Inhibition type ***	Isoelectric point	Hydropathy
**Chen *et al.* (2021)**	GINY (P2)<br>DQW (P8)<br>DQWL (P13)<br>LFQ (P14)	Bovine alpha-lactalbumin	Alpha-lactalbumin fraction 3	UniProt	465.22<br>447.17<br>560,259<br>406,221	NI**	NI	All are stable (NI values)	NI	NI	NI	NI
**Coronado-Cáceres *et al.* (2020)**	EEQR<br>GGER<br>TIAV<br>AGRP<br>VTDG<br>NTQR<br>EQCQR<br>VTDG<br>NQGAI<br>QTGVQ<br>VSTDVNIE<br>HSDDDGQIR<br>SDNE<br>CSTSTV	Cocoa Seeds (*Theobroma cocoa* L.)	**Vincilina :**<br>EEQR<br>GGER<br>TIAV<br>AGRP<br>VTDG<br>NTQR<br>EQCQR<br>VTDG<br>NQGAI<br><br>**Albumin:**<br>QTGVQ<br>VSTDVNIE<br>HSDDDGQIR<br>SDNE<br>CSTSTV	UniProt<br>PeptideCutter<br>MarvinSketch	560.25<br>417.20<br>402.24<br>399.22<br>390.17<br>517.26<br>662.27<br>390.17<br>501.25<br>531.26<br>875.42<br>1041.44<br>463.15<br>463.15	Low (from 8.48 to 17.74 kcal/mol)	NI		Non-toxic	NI	4.08<br>6.85<br>5.52<br>11.18<br>3.13<br>10.6<br>6.16<br>3.13<br>5.36<br>5.35<br>2.87<br>4.20<br>2.87<br>5.25	NI
**Grancieri *et al.* (2021)**	NSPGPHDVALDQ (Pep1)	Chia seed (*Salvia Hispanic* L.)	Gluteline fraction	MarvinSketch	NI	Low (value not informed)	NI	NI	NI	NI	NI	NI

	RMVLPEYE LLYE (P ep2)										
**Ketprayoon *et al.* (2021)**	FYLGYCDY	Defatted rice bran (DORB)	Fraction 5 of DORB through the use of Alcalase ®	Discovery Studio 2019	104.15	High (25%)	Bad	NI	Non toxic	Lineweaver-Burk method)<br><br>Competitive (*docking*)	NI	NI
**Wang *et al.* (2022)**	E.W.<br>NIF<br>AGY<br>PIF<br>QWM<br>TF	Sesame (*Sesamum indicum* L.)	11S globulin and 2S albumin	UniProt ExPASy PeptideCutter	333,344<br>392,456<br>309,322<br>375,469<br>463.56<br>266,297	NI	Good	NI	Non-toxic	EW and AGY (non-competitive)<br><br>NIF, QWM and TF (mixed)<br><br>PIF (NI)	NI	NI
**Xiang *et al.* (2020)**	LR<br>VR<br>APYR<br>DR<br>EEAASLR<br>ELR<br>EWR<br>FLR<br>FMDR<br>FR<br>ALR<br>LLR<br>MR	Sea buckthorn seed flour (*Hippophae rhamnoides*)	Hawthorn seed hydrolyzate identified by HPLC/MS/MS	ChemBio3D	288.2034<br>274.1871<br>527.3198<br>288.2049<br>771.3404<br>419.7802<br>515.3105<br>435.2698<br>591.3614<br>322.1875<br>359.2619<br>401.2879<br>303.2348	NI	NI	NI	NI	Non-competitive	AT	NI

	NLLHR				651.3099							
	PECR				503.3693							
	PR				274.1870							
	QR				303.1838							
	RDR				447.2813							
	SDR				381.2993							
	TR				299.1724							
	W.R.				363.2385							
	WRN				476.3103							
**Zhao *et al* . (2024)**	LGGLDSSLL<br>PH<br>FDTGSSFYN<br>KPAG<br>IWVGGSGM<br>DM<br>YLQGFGKN<br>IL<br>IFNNDPNN<br>HP	Adzuki beans (Vigna angularis)	Fraction 1 (<3 kDa) of adzuki bean protein hydrolyzate	UniProt	1220.6765<br>1389.6201<br>1051.4467<br>1151.6339<br>1180.5261	NI	NI	67.6 (unstable)<br>15.17 (stable)<br>4.36 (stable)<br>6.12 (stable)<br>96.31 (unstable)	Non-toxic	NI	4.98<br>6.66<br>3.11<br>4.98<br>9.78	0.692<br>-0.731<br>0.61<br>-1.66<br>0.19

* A:alanine ; C: cysteine; D: aspartic acid; E: glutamic acid; F: phenylalanine; G: Glycine; H: histidine; I: isoleucine; K: lysine; L: leucine; M: methionine; N: Asparagine; P: Proline; P: glutamine; A: arginine; S: serine; T: threonine; V: valine; Y: Tyrosine; W: tryptophan. **NI= not informed. *** Lineweaver-Burk method.

Table S3. Characteristics of in silico experiments.

Authors/ Year	Peptides/Sequence*	Binding free energy (kcal/mol)	Docking score	Software for molecular docking	In *silico target*	Structure code (PDB)	Mutated structure	Expression organism	Results observed in docking
**Chen *et al.* (2021)**	GINY (P2)<br>DQW (P8)<br>DQWL (P13)<br>LFQ (P14)	-6.53<br>-7.86<br>-4.78<br>-5.11	NI	AutoDock Vina	Peroxisome proliferator-activated receptor alpha (PPARα)	3SP6	No	*Homo sapiens*	P2, P8, P13, and P14 bound to the MET355 residue of the PPARα active site through hydrophobic interactions. P8 formed hydrophobic interactions with other PPARα residues (HIS440, TYR464, TYR314 and SER280) and had lower binding free energy (ELL). Furthermore, P8 has been suggested as the most potent peptide compared to other peptides because it can bind up to six hydrogen bonds.
**Coronado -Cáceres *et al.* (2020)**	EEQR<br>GGER<br>TIAV<br>AGRP<br>VTDG<br>NTQR<br>EQCQR<br>VTDG<br>NQGAI<br>QTGVQ<br>VSTDVNIE	EEQR (−6.5), GGER (−6.3), TIAV (−6), AGRP (−5.9), VTDG (−5.8), NTQR (−5.8), EQCQR (−5.7), VTDG (−5.8),NQGAI (-5.6), QTGVQ (-6.2), VSTDVNIE (-6.1), HSDDDGQIR (-5.9), SDNE (5.7), CSTSTV (-5.5)	NI	AutoDock Vina	Pancreatic triglyceride lipase	1LPB	No	*Homo sapiens*	The EEQR, GGER, QTGVQ and VSTDVNIE peptides showed the highest theoretical affinity with PL and higher than that shown by orlistat . Drug interaction with various amino acids includes Y 288 through hydrogen bonding of carbon interaction, V 232, K 238, D 331 and P 235 through van der Waals interactions , and E 233 and G 236 through alkyl and pi-alkyl interactions . An PL and EEQR

	HSDDDGQIR<br>SDNE<br>CSTSTV	CONTROL:<br>ORLISTAT (-4.3)							interaction could be observed with interactions at K 239, R 265, T 271, N 88, Y 267, N 92, S 333 and D 331, and double bond K 268 presenting van der Waals interactions , carbon- hydrogen and unfavorable donor-donor.
**Grancieri *et al.* (2021)**	NSPGPHDVALDQ (PEP1)<br>RMVLPEYELLYE (PEP2)	PPARy (-5.6)<br>MAGL (-6.4)<br>FAS (-7.3)<br><br>PPARy (6.9)<br>MAG (-7.3)<br>FAS (-5.9)	NI	AutoDock Vina	Peroxisome proliferator - activated receptor gamma (PPARγ)<br><br>Fatty acid synthase (FAS)<br><br>Monoacylglycerol lipase (MAGL)	PPARy (5DSH)<br><br>MAGL (3PE6)<br><br>FAS (2PX6)	3PE6 (Yes)	*Homo sapiens*	The peptides showed interactions with several aas of the enzymes FAS, MAGL, and PPARγ receptor .<br><br>**PPARγ**<br>PEP1= LYS230; ALA235; LYS232; ALA231; TYR219; GLU378; AND ARG234<br><br>PEP2= GLN420; TYR219; LYS224; ILE223; LYS232; THR241; ALA231; ARG234; GLU378; ASP380; HIS425<br><br>**MAGL**<br>PEP1= ARG98; ASP26; SER91; VAL90; VAL95; VAL78; CYS208; ILE211; SER218; LYS160<br><br>PEP2= MET123; SER122; SER155; LEU148; LEU213; ALA151; ALA156; LEU214; LEU150; PRO153; SER218; ARG222;

									LYS160; ALA164; ALA163; ILE211; LEU167; CYS208; and VAL207<br><br>**FAS**<br>PEP1= GLU2227; GLY2228; TYR2288; PRO2229; CYS2292; LYS2436; ASP2291; THR2434; ARG2275; ARG2421; ARG2428; TYR2433; ILE2282; HIS2283; SER2281; LEU2279; AND ASP2280<br><br>PEP2= SER2281; GLU2227; LYS2436; TYR2288; THR2230; PRO2229; AND GLN2432<br><br>PEP2 showed the greatest interaction with PPARγ by decreasing ELL (-6.9 kcal/mol) and with MAGL by also decreasing ELL (-7.3 kcal/mol). Pep1 had the greatest interaction with FAS (−7.3 kcal/mol).
**Ketprayoon *et al.* (2021)**	FYLGYCDY<br><br>Control: Orlistat	NI	122.54	GOLD 5.7.1	Porcine pancreatic lipase (PPL)	1ETH	No	*Sus scrofa*	The amino acid TYR, classified as hydrophobic and<br>located in position 2 of the inhibitory peptide, is capable of<br>binding to Phe216 of PPL. PHE216 acted as the substrate-binding site of PPL, while SER153, ASP177, and HIS264 acted as catalytic sites

									of PPL. The peptide did not bind to these catalytic sites, but was under the influence of van der Waals forces .<br><br>orlistat binds to PPL in the main catalytic sites, SER153 and HIS264, of the PPL complex , the H donor of SER153 binds to the b- lactone ring of orlistat , while HIS264 to the side chain of the aminoester of orlistat , binding, respectively, through a conventional hydrogen bond and Pi-alkyl interaction.<br><br>The peptide had a higher *docking score* (122.54) than orlistat (101.44) with PPL.
**Wang *et al.* (2022)**	E.W.<br>NIF<br>AGY<br>PIF<br>QWM<br>TF	EW (-8.1)<br>NIF (-8.1)<br>AGY (-7.9)<br>PIF (-7.9)<br>QWM (-7.6)<br>TF (-7.4)<br><br>Controls :<br>orlistat -7.7, NLLHR -7.1 PECR -7.3 APYR -7.3, FMDR -7.4, FYLGYCDY -7.9 AIGVGAIER - 6.9	NI	AutoDock Vina	Pancreatic triglyceride lipase	1LPB	No	*Homo sapiens*	The 6 peptides can interact with PHE77, HIS151, SER152, PHE215 and HIS263. Hydrogen bonds are formed between PL and EW, NIF, AGY, PIF, QWM and TF were 3, 3, 4, 1, 4 and 2, respectively. Hydrogen bonding was observed between the carbonyl atoms of the 6 peptides and the P residue HE77, which was reported to be the H donor and electrostatic stabilizer for substrate hydrolysis. EW, AGY, and QWM form hydrogen

									bonds with residue SER152 with bond lengths <3.1 Å. NIF and PIF bind to Ser152 through van der Waals forces . Most peptides interact with residues HIS151 and HIS263 through attractive charges and bind to PHE215 through $\pi-\pi$ stacking interactions . The peptides can enter the hydrophobic pocket of PL and directly bind to SER152 and HIS263 in the catalytic triad of PL to avoid the substrate.
**Xiang *et al.* (2020)**	LR<br>VR<br>APYR<br>DR<br>EEAASLR<br>ELR<br>EWR<br>FLR<br>FMDR<br>FR<br>ALR<br>LLR<br>MR<br>NLLHR<br>PECR<br>PR<br>QR<br>RDR	LR (-5.6)<br>VR (-5.2)<br>APYR (-5.0)<br>DR (-5.4)<br>EEAASLR (-5.5)<br>ELR (-4.5)<br>EWR (-4.8)<br>FLR (-5.6)<br>FMDR (-4.2)<br>FR (-5.4)<br>ALR (5.1)<br>LLR (-4.3)<br>MR (-2.1)<br>NLLHR (-4.8)<br>PECR (-0.4)<br>PR (-6.0)<br>QR (-5.2)<br>RDR (-5.4)	AT	AutoDock Vina	Porcine pancreatic lipase (PPL)	1ETH	No	*Sus scrofa*	The EEAASLR peptides (VAL322, GLN324, GLN188); NLLHR (HIS 224,ASN 320); RDR (SER323, VAL322, GLN324) and VR (PRO194) were the 4 peptides that showed the most interactions with PPL and all had hydrophobic interactions between their aa and hydrophobic residues of PPL. The VR peptide had at least one type of interaction with PPL. The interactions (hydrophilic interactions, hydrogen bonds, hydrophobic interactions, π - π interaction/stacking, and Van der Waals interactions) could stabilize the peptide-PPL complex and/or affect the PPL conformation or

	SDR<br>TR<br>W.R.<br>WRN	SDR (-4.8)<br>TR (-5.2)<br>WR (-4.3)<br>WRN (-4.7)							cause conformational change to accommodate the substrate. Hydrogen bonding has been speculated to be a major contributor to the PPL inhibition offered by inhibitory peptides containing an ARG residue. ARG contains an α-amino group, an α-carboxylic acid group and a side chain containing a guanidino group , thus being able to interact with both charged residues through hydrogen bonds and to interact with hydrophobic residues through hydrophobic interaction through its methylene group.
**Zhao *et al* . (2024)**	LGGLDSSLLPH<br>FDTGSSFYNKPAG<br>IWVGGSGMDM<br>YLQGFGKNIL<br>IFNNDPNNHP	**TO PL=**<br>LLGGLDSSLLPH (−126.7088)<br>FDTGSSFYNKPAG (−121.9676)<br><br>IWVGGSGMDM (−121.7003)<br><br>YLQGFGKNIL (−118.7321)<br><br>IFNNDPNNHP (-116.5371)	AT	Dock	PPL<br><br>CE	1ETH<br><br>1F6W	1F6W Yes	*Sus scrofa*<br><br>*Homo sapiens*	The five peptides interacted with 7 to 11 amino acid residues, indicating a close association with lipase, among which IWVGGSGMDM and LLGGLDSSLLPH could bind to 7 and 11 amino acid residues of lipase, respectively. LLGGLDSSLLPH, FDTGSSFYNKPAG, and IFNNDPNNHP not only have the ability to establish hydrogen bonds and salt bridges with lipase catalytic residues, specifically

**FOR CE=**<br>LGGLDSSLLPH (-139.0741)<br>FDTGSSFYNKPAG (−148.9364)<br><br>IWVGGSGMDM (−132.9048)<br><br>YLQGFGKNIL (−132.1326)<br><br>(IFNNDPNNHP (-132.1017)	SER153 and HIS264, but can also interact with substrate-binding residues. IWVGGSGMDM and YLQGFGKNIL do not bind to catalytic lipase residues, but can bind to substrate-binding residues through hydrophobic interactions. Most peptides also indicated the ability to bind to other lipase residues (ALA261, PHE259 , and VAL260) that are not characteristic catalytic or substrate-binding sites of lipase, which may indicate indirect PL inhibitory activity.<br><br>The 5 antiobesity peptides interacted with 7 to 16 amino acid residues of CE, among which IFNNDPNNHP and FDTGSSFYNKPAG bound to 7 and 16 amino acid residues, respectively. The interactions between the five peptides and the amino acid residues of the CE encompassed hydrogen bonds, hydrophobic interactions, and salt bridges. LLGGLDSSLLPH, FDTGSSFYNKPAG and IWVGGSGMDM inhibit CE activity by occupying both the catalytic site (S ER194 and HIS435)

	and substrate binding sites (ALA108), while YLQGFGKNIL inhibits CE activity by binding to the catalytic site, and IFNNDPNNHP inhibits CE activity binding to substrate binding sites .

* A:alanine ; C: cysteine; D: aspartic acid; E: glutamic acid; F: phenylalanine; G: Glycine; H: histidine; I: isoleucine; K: lysine; L: leucine; M: methionine; N: Asparagine; P: Proline; P: glutamine; A: arginine; S: serine; T: threonine; V: valine; Y: Tyrosine; W: tryptophan. NI= not informed.
